# An easy and inexpensive method for determining the rate of individual phoretic events of nematodes

**DOI:** 10.17912/micropub.biology.000942

**Published:** 2023-09-21

**Authors:** Benjamin Williams, Matthew Nelson, Scott McRobert, Jonathan Fingerut

**Affiliations:** 1 Biology, Saint Joseph's University, Philadelphia, Pennsylvania, United States

## Abstract

Considering their limited locomotory capabilities, the cosmopolitan distribution of free-living nematodes may rely on phoretic dispersal. We describe a new, inexpensive device to investigate individual phoretic events of the nematode *Caenorhabditis elegans* using the pomace flies *Drosophila melanogaster* and *Drosophila hydei* over short time periods. Using our device, we replicated previous findings demonstrating that phoresis requires *C. elegans* to be in the dauer stage and capable of nictation. Additionally, we find that phoresis can happen on the order of seconds, and does not increase linearly with time of interaction. Using this approach can facilitate the investigation of nematode biogeography, which could provide useful insight into their, and their vector’s, control.

**Figure 1.**
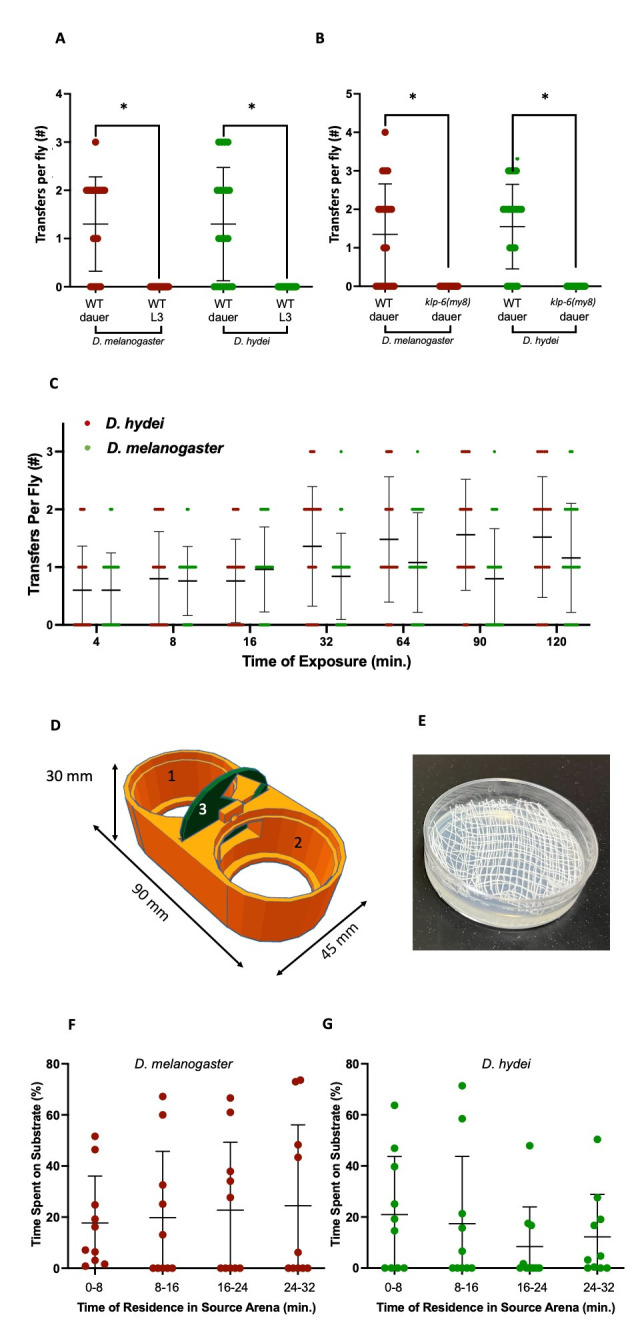
**A.** Transfer per fly for wild-type dauers and wild-type L3 stage worms after 30 minutes of contact. Error bars represent the standard deviation, middle bar represents the average. Each dot represents an individual fly (n=20). * denotes a p-value of less than 0.001 from Mann-Whitney U test. **B.** Transfers per fly for wild-type and *klp-6* ( *my8* ) dauers. Error bars represent the standard deviation and the middle bar represents the average. Each dot represents an individual fly (n=20). * denotes a p-value of less than 0.001 from Mann-Whitney U test. **C.** Transfers per fly following increasing contact time between dauers and flies. Error bars represent the standard deviation, middle bar represents the average. Each dot represents an individual fly (n=20). Dots are reduced in size to allow for overall visual clarity (n=25 for each time point). Therefore, the width of those lines of dots represents the relative number of flies with that level of transfer at each time point. **D.** Rendering of 3D-printed transfer chamber 1: source arena, 2: sink arena, 3: dividing gate in closed position. **E.** Source dish with cheese cloth to provide nictation substrate. **F.** Percentage of time *D. melanogaster* spent in contact with the substrate over time. Error bars represent the standard deviation, middle bar represents the average. Each dot represents an individual fly (n=10). **G.** Percentage of time *D. hydei* spent in contact with the substrate over time. Error bars represent the standard deviation, middle bar represents the average. Each dot represents an individual fly (n=10).

## Description

The geographic distribution of an organism is defined by both the site of, and its ability to reach, a suitable habitat. For many organisms it may be the latter that is the major limiting factor, due to physical barriers ( *e.g.,* bodies of water, topography) or the distance between locations. Some movement-challenged organisms such as windborne seeds (Ran et al., 2008) and marine larvae (Fonseca, 1999) disperse by riding passively on fluid flow. Others, such as trematodes, have developed parasitic life cycles where they are carried inside hosts over distances orders of magnitude greater than they could otherwise travel (Smith, 2001) . Some, however, use a third strategy, relying on a commensal relationship with the organisms that carry them.

Despite their small size and limited locomotion, free-living nematodes such as *Caenorhabditis elegans* have a remarkably cosmopolitan distribution (Frézal and Felix, 2015). It is therefore likely that phoresis, the transient attachment to another organism for the purposes of transport, may be an important part of their, and many other nematodes’ ecology. In fact, phoretic relationships have already been documented with beetles (Kruitbos et al., 2009) , wasps (Gupta et al., 2021) , isopods (Lee et al., 2017) , and flies (Lee et al. 2011) . Nictation behavior while in the worm’s dauer stage appears to be critical in facilitating phoresis (Lee et al., 2011) . The dauer stage replaces the normal L3 stage under certain adverse conditions ( *e.g.,* overpopulation, starvation, high temperature) in some nematodes ( *C. elegans* among them) (Cassada & Russel, 1975) . Nictation is a behavior exhibited by dauers where the anterior part of their body is raised and waved in three-dimensional spirals and loops. This is thought to increase contact rates with potential carriers (Campbell & Gaugler, 1993) .

To date, investigations of nematode phoresis have primarily studied groups of hosts at the temporal resolution of, or greater than, a day (Rinker and Bloom, 1982, Lee et al., 2011, Lee et al., 2017) . This study illustrates the efficacy of a new technique that facilitates studying individual hosts at temporal scales of minutes and allows individual phoretic events (transfer between source and sink) to be isolated using a purpose-designed 3D-printed chamber that can be easily produced with consumer-grade printers at low cost.

Using this new technique, we were able to replicate previous findings (Lee et al., 2011) regarding the critical role of nictation behavior in worm phoresis, and add to the understanding of the timing of this phenomena. Further, we were able to directly compare phoresis via two different pomace fly species ( *Drosophila melanogaster* and *Drosophila hydei* ) only one of which ( *D. melanogaster* ) appears to have been studied previously in terms of its role in phoresis. In both species, the dauer stage was required for transfer, since no L3 worms were transferred phoretically via either species of fly, while dauers were consistently carried by both species ( **Figure 1A** ). To determine if nictation was required or just the dauer morphology, we measured phoresis in *klp-6* mutants, who display defective cilia in the IL2 neurons (Morsci & Barr, 2011) and altered nictation behavior (Lee et al., 2011) . As with the L3, zero transfers were observed using these mutants ( **Figure 1B** ), suggesting a requirement of nictation for phoresis.

The ability of the new design to evaluate phoresis at small temporal scales supports previous work (Lee et al. 2011) , indicating that transfer occurs at a very low level. Additionally, we find that transfer occurs quickly (< 4 minutes of contact between fly and worm) but that more time of exposure does not necessarily lead to more transfer ( **Figure 1C** ). Transfer levels leveled off starting at 32 minutes for *D. melanogaster* (1.52 ± 1.03, average ± S.D.) and after 16 minutes for *D. hydei* (1.01 ± 0.89). Comparisons between time-points within species were made using a Mann-Whitney U test as the data was non-normal. *D. hydei* showed no significant differences except between the 4 and 120-minute time points, while *D. melanogaster* separated into two distinct ranges within which no significant difference was seen (4-16 min and 32-120 min) but all comparisons between those two ranges was significant (p<0.05). Comparison between species at each time point found no significant difference except at 90 minutes (z=-2.62, p = 0.01). The reason for the low rate of transfer and the leveling off is unknown but may be influenced by the flies’ frequent grooming (Mueller et al., 2022) .

Even though the transfer rate may be low, phoretic dispersal of nematodes by flies might be quite common in nature given the sheer number of both in the environment, and their overlapping presence on rotting fruit. Using our technique to explore these mechanisms may be useful in understanding the ubiquity of nematodes given their limited capacity for movement. Furthermore, the identification of suitable nematode species could possibly assist in controlling some agriculturally important pest fly populations, as nematode phoresy has been shown to have negative impacts on at least one other vector species (Giblin-Davis et al., 2013, Wang and Rozen, 2018) .

## Methods

Fly Cultures

Experiments were conducted using mass bred *D. melanogaster* and *D. hydei* maintained at 25 ± 1℃ in uncrowded conditions, housed in 200 ml stock bottles with standard fly media (Carolina Formula 4-24), plus brewer’s yeast. Flies were transferred under CO _2_ anesthesia.

*C. elegans* Husbandry

Worms were cultivated at 20℃ on standard NGM medium (1.7% agar) and fed the OP50 derivative *E. coli* strain DA837 (Davis et al., 1995) . Synchronized L3 larvae were isolated following the bleaching egg prep method (Steirnagle, 2006). Bleached eggs were allowed to hatch overnight in M9, L1 worms were plated the following day, and worms were collected when they reached the L3 stage. Dauer worms were collected from multiple plates that were left for 7-9 days to induce starvation. Dauers were isolated from the other larvae on the plates using the Sodium Dodecyl Sulfate (SDS) protocol (Cassada & Russel, 1975) , in which they were washed from the plates and incubated in 1% SDS for 30 minutes.

Quantifying Phoresis

We designed a 3D-printed chamber (the .stl file can be found in the extended data and printed for ~$10 per unit via online production houses such as https://www.voodoomfg.com if a printer is not available) that allowed a single fly to make contact with a Petri dish containing worms in a source arena. After a set period of time the fly could be moved, without contact, to an adjacent sink arena containing an agar-only Petri dish where the worms could detach and be counted ( **Figure 1D** ). The 35 mm plastic Petri dishes fit snugly inside and formed the bottom of the 37 cm ^3^ cylindrical arenas. The source dishes were filled with standard NGM/agar, covered with a layer of cheese cloth to provide a substrate for the worms to nictate off of, as previously described (Lee et al. 2011; Lee et al., 2017, **Figure 1E** ), and then were seeded with ~1000 worms. A fly was transferred via suck tube into the source arena with the gate between arenas rotated to the closed position, after which the lid of the Petri dish was placed over the top to contain the fly but still allow for its observation. The rotating gate allowed for the connection between arenas to be easily opened and closed without allowing flies to escape the chamber. After the prescribed amount of time the gate was rotated to align its opening with the tunnel between the arenas and the fly was allowed to move to the other side, after which the gate was rotated closed behind it. In the rare cases where the fly did not move on its own, a flashlight shone into the source arena encouraged the fly to quickly move over. Flies were left in the sink arena for 10 minutes, after which the fly was carefully removed via suck tube and the dish placed under a dissecting microscope to determine the number of worms that had been transferred. A new dish and fly was used for each trial. Controlling movement between source and sink plates allowed us to study individual phoretic events, directly associating any worms found on the sink plate to the movement of a single fly. All worms on the sink dish could be attributed to phoretic transfer via flies since controls with no flies and the gate open for 24 hours showed no worms present in the sink arena (n=10).

In order to determine the rate at which flies could be transferred and the impact of contact time on that rate, we first had to know if the length of time spent in the arena influenced the amount of time the fly spent on the dish where contact could take place. Individual flies (n=10) were observed over four consecutive eight minutes periods after they entered the source arena, and the % of time the fly was in contact with the arena floor was determined for each time period. Neither fly species’ time of residence in the source arena had a significant impact on the time of potential contact with the worms (one-way repeated-measures ANOVA, df = 3, F = 0.82 p = 0.49 for *D. melanogaster* , and df = 3, F = 0.2, p = 0.89 for *D. hydei* ) ( **Figure 1F,G** ). During their total time in the arena, flies spent 15.5% ± 5.5% for *D. melanogaster* and 21.2% ± 3.0% (average and S.D.) for *D. hydei* walking or standing on the source dish. No statistical difference was found between species (one-way ANOVA df = 1, F = 1.58, p = 0.21). The lack of a significant change over time in the flies’ behavior (time spent on the plate) meant that time in the chamber reflected time of potential contact in a linear fashion. For the time trials, flies were left in the source arena with dishes of wild-type dauers for 4, 8, 16, 32, 64, 90 and 120 min (n=25 for each treatment) before being allowed to move to the sink arena.

## Reagents

*Flies*

*D. melanogaster* and *D. hydei* stocks were derived from flies collected in Bryn Mawr, Pennsylvania.

*Nematodes*

The worm strains used in this study were: N2 ( *wild type* ) and PT1194 *klp-6* ( *my8* ); *him-5* ( *e1490* )

*Bacteria*

OP50 derivative *bacteria E. coli* strain DA837

## Extended Data

Description: .stl file for 3D printing the phoresis chamber. Resource Type: Model. DOI: 10.22002/q6bjx-ybr45
